# Flight aerodynamics in enantiornithines: Information from a new Chinese Early Cretaceous bird

**DOI:** 10.1371/journal.pone.0184637

**Published:** 2017-10-11

**Authors:** Di Liu, Luis M. Chiappe, Francisco Serrano, Michael Habib, Yuguang Zhang, Qinjing Meng

**Affiliations:** 1 University of Chinese Academy of Sciences, Beijing, China; 2 Beijing Museum of Natural History, Beijing, China; 3 Dinosaur Institute, Natural History Museum of Los Angeles County, Los Angeles, California, United States of America; 4 Universidad de Málaga, Campus Universitario de Teatinos s/n., Málaga, Spain; 5 The University of Southern California, Los Angeles, California, United States of America; University of Akron, UNITED STATES

## Abstract

We describe an exquisitely preserved new avian fossil (BMNHC-PH-919) from the Lower Cretaceous Yixian Formation of eastern Inner Mongolia, China. Although morphologically similar to Cathayornithidae and other small-sized enantiornithines from China’s Jehol Biota, many morphological features indicate that it represents a new species, here named *Junornis houi*. The new fossil displays most of its plumage including a pair of elongated, rachis-dominated tail feathers similarly present in a variety of other enantiornithines. BMNHC-PH-919 represents the first record of a Jehol enantiornithine from Inner Mongolia, thus extending the known distribution of these birds into the eastern portion of this region. Furthermore, its well-preserved skeleton and wing outline provide insight into the aerodynamic performance of enantiornithines, suggesting that these birds had evolved bounding flight—a flight mode common to passeriforms and other small living birds—as early as 125 million years ago.

## Introduction

The clade Enantiornithes includes the most diverse (taxonomically and ecologically) group of Cretaceous birds [1]. In the past three decades, more than 40 enantiornithine species have been named from the Early Cretaceous Jehol Biota (northeastern China) alone, and the pace of new discoveries continues unabated [2–5].

**Fig 1:**
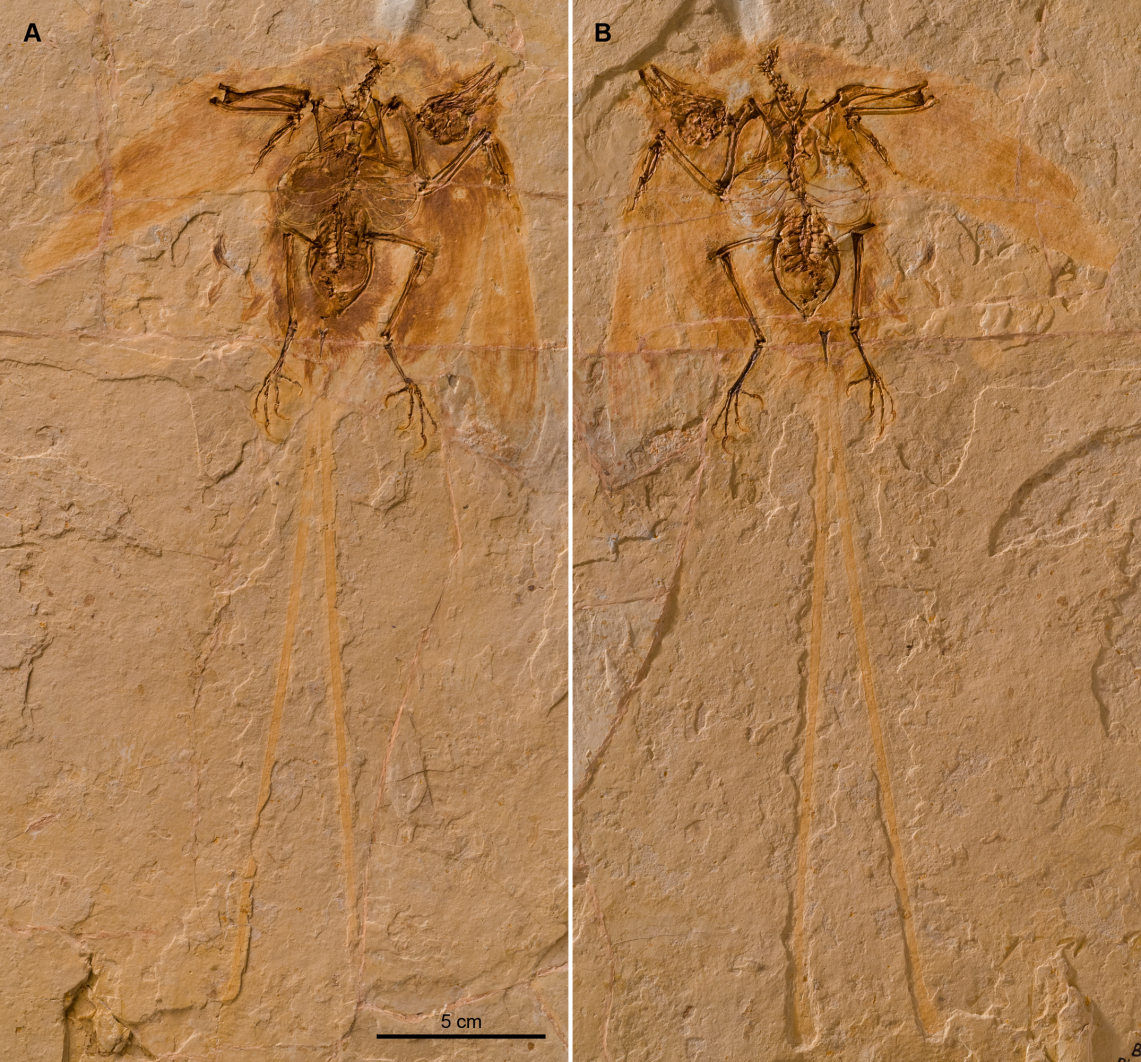
Slab (BMNHC-PH 919a) (A) and counterslab (BMNHC-PH 919b) (B) of *Junornis houi*.

Many of these exceptionally well-preserved avian fossils reveal details of their soft-tissues, which have allowed interpretations about various aspects of their biology [6,7]. Nonetheless, our understanding of the flight performance evolved by these birds remains limited. Here we describe a new avian fossil (BMNHC-PH 919) from the Yixian Formation of eastern Inner Mongolia (northeastern China), which represents another addition to the enantiornithine diversity of the Jehol Biota. Most importantly, the well-preserved skeleton and wing outline of this new fossil (Fig 1) allows us to infer specific aspects of its aerodynamic performance, thus shedding light on the flight styles of some of the earliest avian lineages.

Furthermore, the new specimen joins a handful of Early Cretaceous fossils discovered in Inner Mongolia. The first of these rare fossils was identified as the holotype of the enantiornithine *Otogornis genghisi* (IVPP V9607) [8], collected from the Jingchuan Formation (contemporaneous to the Jehol’s Jiufotang Formation) at Chabu Sumu, west of Otog Qi (Yike Zhaomeng) in western Inner Mongolia. Subsequently, Li *et al*. (2008) [9] reported on another enantiornithine specimen (BMNHC-PH110) from the same site and formation, and erected the species “*Cathayornis chabuensis”*(although Wang and Liu, 2015 considered this species as a *nomen dubium*) [10]. More recently, Zhang *et al*. (2010) described another enantiornithine bird (OFMB-3) from the same locality and strata, although these authors refrained from naming it [11]. While contemporaneous with the Jehol sites of northeastern China, these previous occurrences are hundreds of kilometers away from the localities of this celebrated biota. The new specimen, however, is from strata clearly identified as belonging to the Jehol Biota. The geographic provenance of BMNHC-PH 919, the first Jehol enantiornithine described from eastern Inner Mongolia, adds new information for understanding the distribution of the Jehol enantiornithines, which prior to this discovery were restricted to the Liaoning and Hebei provinces.

## Materials and methods

The new specimen, virtually complete and preserved in two slabs (Fig 1), was discovered in the Yixian Formation, Ningchen County, Inner Mongolia Autonomous Region, China. The staff from the Natural History Museum of Los Angeles County and the Beijing Museum of Natural History prepared the specimen; it is housed at the latter institution and catalogued as BMNHC-PH 919 (a, b).

We estimated the body mass (*BM*), wingspan (*B*), and lift surface (*SL*) of BMNHC-PH 919 following the multivariate approach (and database) of Serrano et al. [12, 13]. Tables 1 and 2 show the measurements and equations used to calculate *BM*, *B*, and *SL* of BMNHC-PH 919, respectively. From these values we calculated the wing loading (*WL = BM/SL*) and aspect ratio (*AR = B*^*2*^*/SL*) of BMNHC-PH 919, the primary aerodynamic parameters used to infer the flight properties of this specimen (see Discussion). The dataset used to construct the morphospace of flight modes in living Neoaves was obtained from ref. [13].

**Table 1:** Selected lengths (mm) of the skeletal elements and structures of *Junornis houi* (BMNHC-PH-919a).

Skull	30.6	Ischium	15*
Coracoid	14.1	Ilium	15*
Scapula	20.1	Pubis	25.5
Furcula	9.5	Pygostyle	12.2
Sternum	18.5	Femur	23.4
Humerus	25.9	Tibiotarsus	28.1
Deltopectoral crest	9.7	Metatarsal II	15.5
Bicipital crest	3.0	Metatarsal III	16.8
Ulna	26.9	Metatarsal IV	16.1
Radius	25.7	Pedal digit I-1	4.3
Radius mid-shaft	1.0	Pedal digit I-2	4.8
Alular metacarpal	1.9	Pedal digit II-1	3.1
Major metacarpal	11.7	Pedal digit II-2	4.8
Major metacarpal mid-shaft	0.9	Pedal digit II-3	5.1
Minor metacarpal	12.6	Pedal digit III-1	4.6
Alular digit-1	4.5	Pedal digit III-2	4.1
Alular digit-2	2.8	Pedal digit III-3	4.5
Major digit-1	5.5	Pedal digit III-4	6.1
Major digit-2	4.1	Pedal digit IV-1	2.8
Major digit-3	2.5	Pedal digit IV-2	2.8
Minor digit-1	3.5	Pedal digit IV-3	2.8
Minor digit-2	1.1	Pedal digit IV-4	3.1
Secondary feather at wrist	71.4	Pedal digit IV-5	5.1
Longest primary feather	72.7	Ornamental rectrix	200

* indicates estimated values.

**Table 2:** OLS multiple regressions used to estimate the body mass (*BM*), wingspan (*B*), and lift surface (*SL*) of BMNHC-PH-919 (see Serrano et al. 2015, 2017).

Variable estimated	Multiple regression (log Y = log a + b1 log X1 + b2 log X2…+ bp log Xp)	N	R^2^_adj_
*BM*	log BM = −0.582 + 1.19 log HL + 0.257 log dpL + 0.574 bcL + 1.861 log UL − 2.944 log RL + 0.645 log dCmW + 0.282 log FL + 1.193 log TL − 0.593 log TmL	407	0.974
*B*	log B = 0.975 + 0.262 log HL+ 0.069 log UL + 0.203 log CmL + 0.444 log Lprim	143	0.992
*SL*	log SL = −4.866 + 0.214 log HL + 0.263 log RL + 0.242 log DCmW + 0.325 log Lprim + 0.951 log c	138	0.991

In regards to the clade Aves, we follow the node-based phylogenetic definition of L. M. Chiappe [14] in which this clade is defined as the common ancestor of *Archaeopteryx lithographica* and Neornithes plus all its descendants; under this definition, the term ‘bird’ is interchangeable with Aves. We also follow P. Sereno’s [15] stem-based definition of Enantiornithes, in which this clade is defined as all taxa closer to *Sinornis santensis* than to Neornithes.

All necessary permits were obtained for the described study, which complied with all relevant regulations. These were obtained from the Chinese government through the Beijing Museum of Natural History. All fossils and specimens collected by the Beijing Museum of Natural History, where the specimen is housed, conform to Chinese regulations.

Institutional Abbreviations: BMNHC, Beijing Museum of Natural History, Beijing, China; IVPP, Institute of Vertebrate Paleontology and Paleoanthropology, Beijing, China; OFMB, Otog Field Museum of Geological Vestiges, Otog Banner, Inner Mongolia Autonomous Region, China.

### Nomenclatural acts

The electronic edition of this article conforms to the requirements of the amended International Code of Zoological Nomenclature, and hence the new names contained herein are available under that Code from the electronic edition of this article. This published work and the nomenclatural acts it contains have been registered in Zoo Bank, the online registration system for the ICZN. The Zoo Bank LSIDs (Life Science Identifiers) can be resolved and the associated information viewed through any standard web browser by appending the LSID to the prefix "http://www.zoobank.org/". The LSID for this publication is: urn:lsid:zoobank.org:act:7388BEC3-33A0-4B21-A67A-AA92B7820E0D. The electronic edition of this work was published in a journal with an ISSN, and has been archived and is available from the following digital repositories: PubMed Central, LOCKSS.

## Results

### Systematic Paleontology

Aves Linnaeus, 1758 [16]

Pygostylia Chiappe, 2001 [17]

Ornithothoraces Chiappe, 1995 [18]

Enantiornithes Walker, 1981 [19]

*Junornis houi* gen. et sp. nov.

urn:lsid:zoobank.org:act:4A0ACE81-17F9-4EA5-AAA6-0FEFE1E1FFAE

### Holotype

A nearly complete and articulated skeleton (BMNHC-PH 919; Beijing Museum of Natural History) contained in two slabs (a, b). While the skeleton is preserved as voids of the bony elements, it is surrounded by feather impressions defining the surface of its wings and body outline (Fig 1).

### Horizon and locality

Yixian Formation, Early Cretaceous (~ 126±4 mya) [20]; Liutiaogou Village, Daming Town, Ningchen County, Inner Mongolia Autonomous Region, China.

### Etymology

The generic name *Jun* is derived from a Chinese character (俊) meaning beautiful; *ornis* is Greek for bird. The species name, *houi* honors Dr. Hou Lianhai and his important contributions to Chinese paleornithology.

### Diagnosis

A small *Cathayornis*-like bird distinguishable from other similar enantiornithines by the following combination of characters: rounded craniolateral corner of sternum (more angular in *Cathayornis yandica* and *Houornis caudatus*); distinct trough excavating ventral surface of mediocranial portion of sternum; triangular process at base of sternal lateral trabecula (absent in *H*. *caudatus* and *E*. *walkeri*); sternal lateral trabecula broad (much wider than in *C*. *yandica*, *E*. *walkeri*, and *H*. *caudatus*) and laterally deflected (straight in *C*. *yandica* and *E*. *walkeri*); sternal intermediate trabecula nearly level with mid-shaft of lateral trabecula (significantly shorter in *C*. *yandica*, *H*. *caudatus* and *E*. *walkeri*); sternal xiphoid process level with lateral trabeculae (trabeculae project further caudal in *H*. *cautus* and *C*. *yandica*); costal processes of last two penultimate synsacral vertebrae three times wider than same process of last synsacral vertebra; and very broad pelvis.

### Anatomical description

Anatomical nomenclature primarily follows [21], using the English equivalents of the Latin terms.

#### Skull

**Fig 2:**
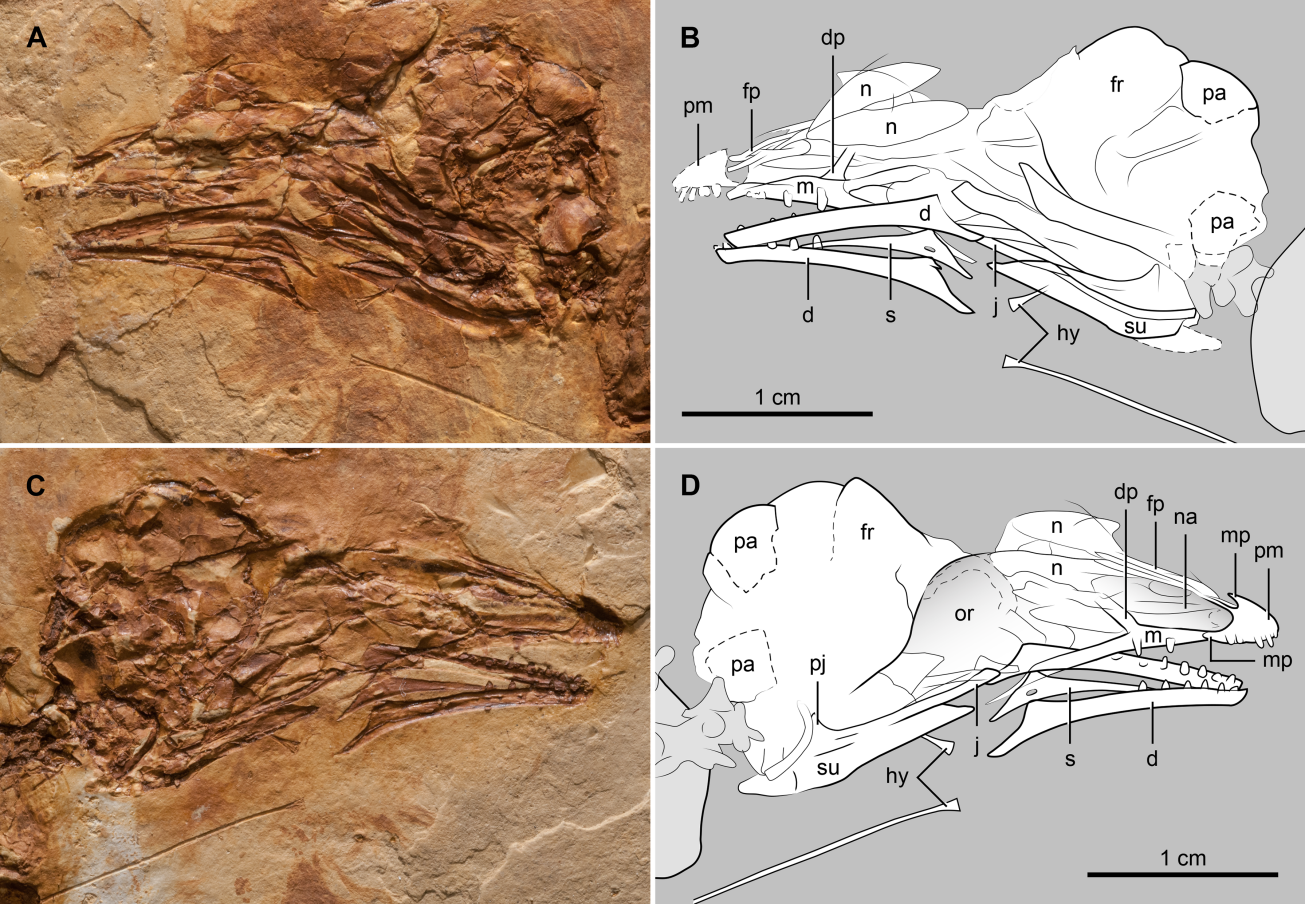
Photograph and interpretive drawing of the skull of *Junornis houi*. (A, B) BMNHC-PH 919a and (C-D) BMNHC-PH 919b. Abbreviations: d, dentary; dp, dorsal process of maxilla; fp, frontal process of premaxilla; fr, frontal; hy, hyoid; j, jugal; m, maxilla; mp, maxillary process of premaxilla; n, nasal; na, external nares; or, orbit; pa, parietal; pj, postorbital process of jugal; pm, premaxilla; s, splenial; su, surangular.

As with the rest of the skeleton, while the skull is mostly articulated, it is largely preserved as a void, thus making only few details discernable (Fig 2). The premaxilla bears four teeth; the first two are clearly peg-shaped. The maxillary process of the premaxilla appears relatively short and caudally pointed, as in *Eocathayornis* walker, *Cathayornis yandica*, and *Houornis caudatus* [11, 22–24]. The frontal process of the premaxilla appears to be close to three times the length of the bone’s maxillary process, a proportion similar to that of *H*. *caudatus* and *C*. *yandica*. On slab ‘b’, the ends of the frontal processes appear to be separated by a suture, thus indicating that these processes were caudally individualized (Fig 2C and 2D). The maxilla is toothed. A short dorsal process defines the caudal margin of the elliptical external nares. Caudal to the dorsal process of the maxilla there is evidence of an antorbital fossa, although its morphology cannot be determined. As in most enantiornithines [2], the nasals are broad and apparently unfused medially (Fig 2). The frontals also appear unfused to the vaulted parietals, as in many Mesozoic birds. The dentaries are not fused rostrally, as in most other enantiornithines [2]. Their dorsal and ventral margins are parallel for most of their lengths becoming slightly divergent caudally, where the dentary broadens and forms a caudoventrally-slanted contact for the post-dentary bones (Fig 2). The right splenial is disarticulated from its dentary; it is a triangular bone with an elongated foramen directly below the apex of the triangle (Fig 2). The rostral branch of the splenial, in front of the foramen, is close to three times as long as the caudal branch. Six and seven teeth are preserved in the right and left dentary, respectively. Their morphology is similar to that of the upper teeth. A pair of very long and rod-like elements of the hyoid apparatus is preserved, presumably corresponding to the ceratobranchial. The length of this bone is nearly half that of the skull, a proportion larger than in most other enantiornithines.

#### Axial skeleton

**Fig 3:**
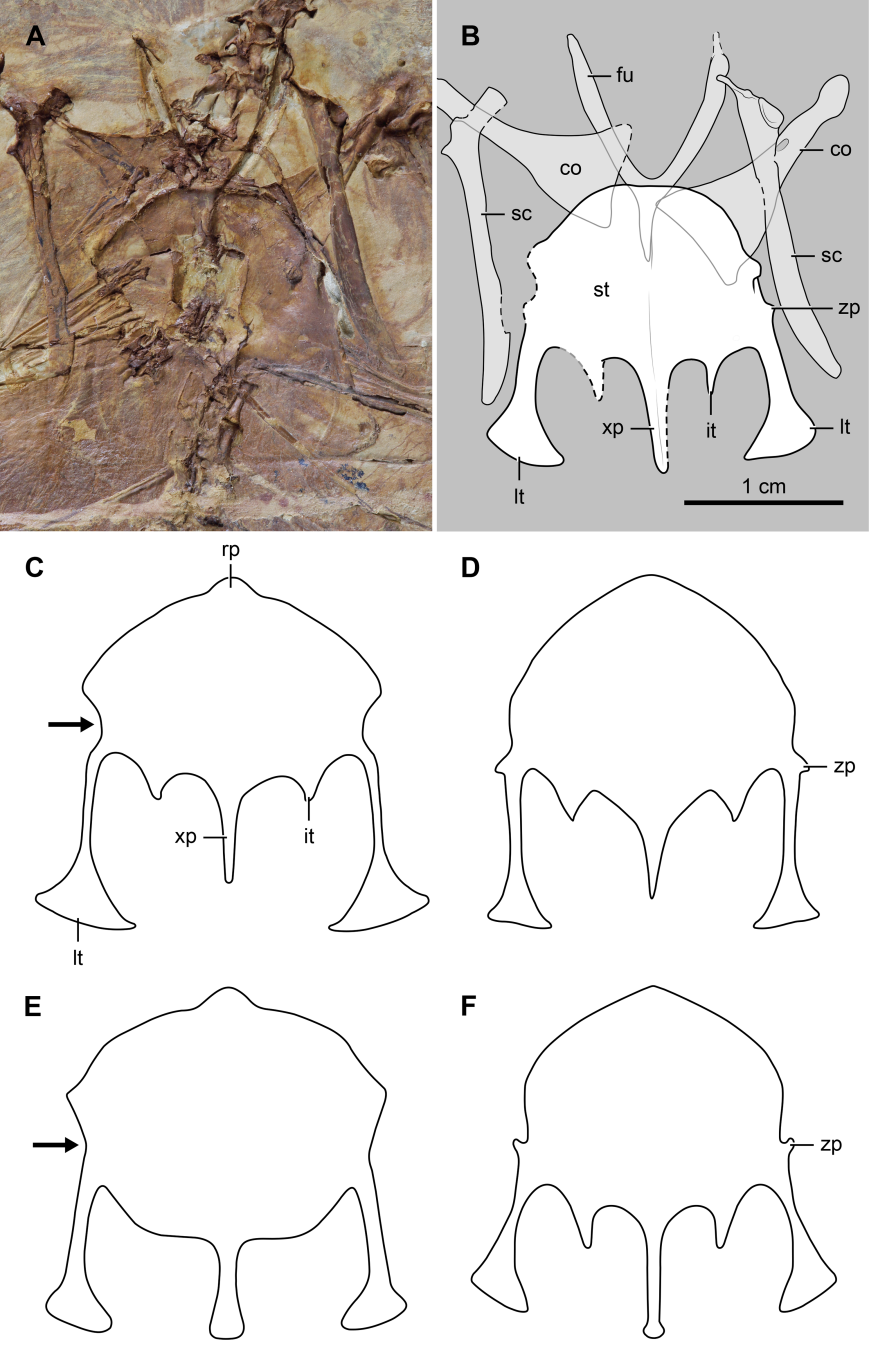
Comparison of the sterna of selected enantiornithines. (A, B), photograph and interpretative drawing of *Junornis houi*, BMNHC-PH919a; (C), *Houornis caudatus*; (D), *Cathayornis yandica* (IVPP 9769); (E), *Eocathayornis walkeri* (IVPP 10916); (F), “*Cathayornis chabuensis*” (OFMB-3). Abbreviations: co, coracoid; fu, furcula; it, intermediate trabecula; lt, lateral trabecula; rp, rostral process; sc, scapula; st, sternum; xp, xiphoid process; zp, zyphoid process. The black arrow in C and E points at the concave costal margin of the sternum of *Houornis caudatus* and *Eocathayornis walker*. Drawings C-F are not to scale.

**Fig 4:**
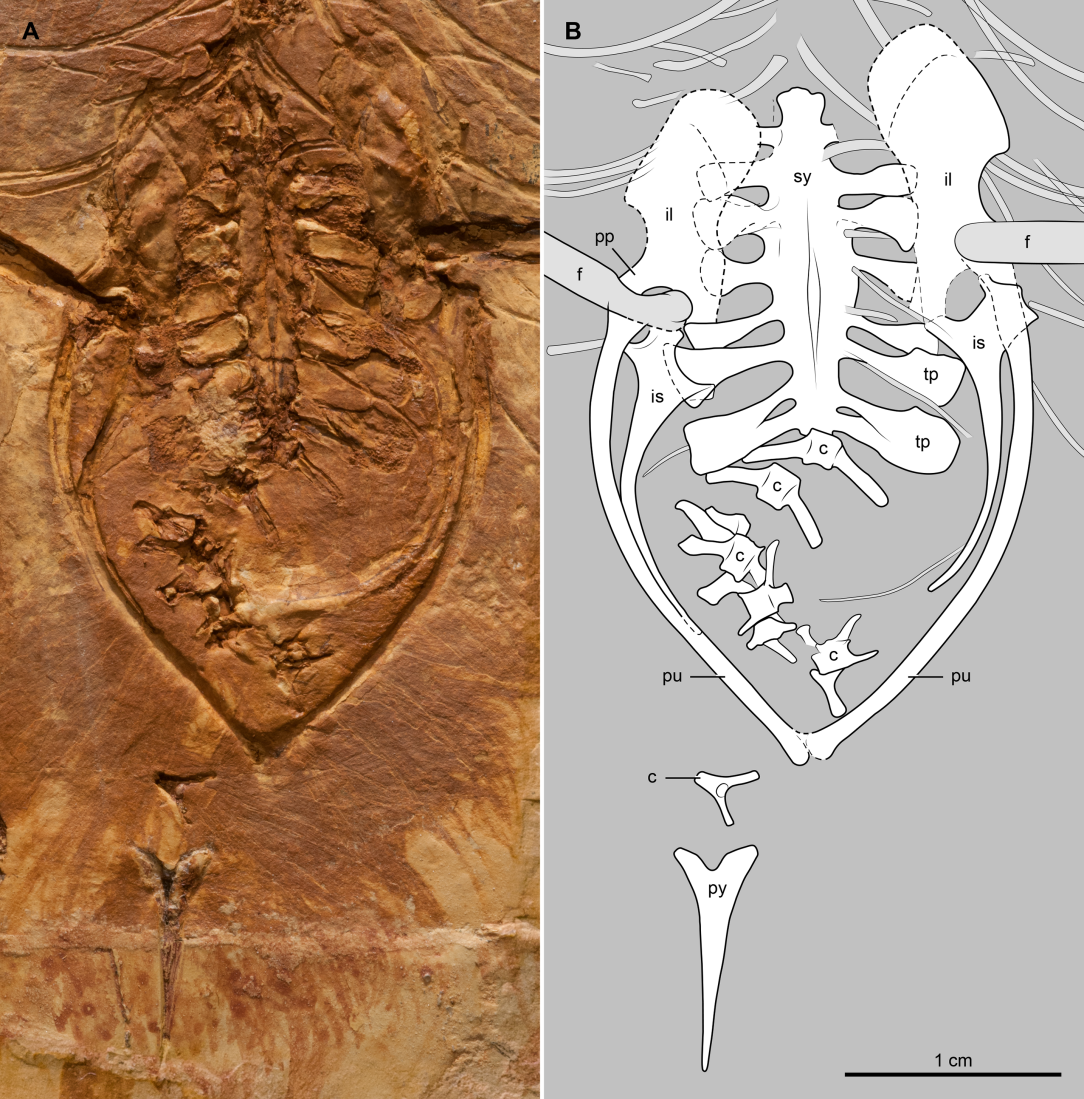
Photograph and interpretative drawing of the pelvic girdle, synsacrum, and caudal vertebrae of *Junornis houi* (BMNHC-PH919a). Abbreviations: c, free caudal vertebrae; cp, costal processes of synsacral vertebrae; f, femur; il, ilium; is, ischia; pu, pubis; py, pygostyle; pp, pubic peduncle of ilium; sy, synsacrum; tp, transverse process.

The vertebral column is incomplete and partially articulated, preserving very few morphological details. There are eight or nine vertebrae of cervical morphology (those that carry short ribs), including the atlas and axis. The sternum and other bones cover most of the anterior thoracic vertebrae; they are best exposed in the posterior region of the series. The neural spines of the middle and posterior thoracic vertebrae are broad and dorsally expanded (Fig 3A) as in *Cathayornis yandica* (in *Eocathayornis walkeri* the neural spines are nearly square-shaped). The cranial and caudal articular facets of these vertebrae are amphicoelous; their parapophyses are centrally located and their centra are excavated by a shallow, longitudinal groove (Fig 3A). There appears to be seven vertebrae in the synsacrum (Fig 4), a number common among enantiornithines [25–26]. A longitudinal sulcus on BMNHC-PH-919a suggests the presence of a dorsal crest running the length of the synsacrum. The costal processes of these vertebrae expand strongly along their distal half. Anteriorly, these processes are as long as the width of the centra; posteriorly, they extend for more than twice the width of the centra (Fig 4). Furthermore, the distal half of the penultimate two processes (corresponding to the 5^th^ and 6^th^ synsacral vertebrae) are extremely expanded, reaching nearly 3 times the width of the process belonging to the last synsacral vertebra. The caudal series is largely disarticulated; at least seven free caudal vertebrae can be discerned (Fig 4). The two most proximal caudals bear long and slender transverse processes that are slightly caudolaterally directed. The morphology of the subsequent free caudals is less clear, although the last one shows the presence of ventrolaterally directed transverse processes. The pygostyle is relatively short (approximately 72% the length of the tarsometatarsus) and proximally forked. The main body of this bone is slender, tapering caudally (Fig 4) and lacking the distal constriction of some other enantiornithines (e.g., *Rapaxavis pani* [25]).

#### Thoracic girdle and sternum

The furcula is Y-shaped as in many other enantiornithines [1]; its interclavicular angle is approximately 55 degrees (Fig 3), broader than in *Sinornis santensis* (50 degrees) [27]. The length of the hypocleidium cannot be determined with precision. Nonetheless, it appears to be close to half the length of the furcular rami. The furcular ramus is L-shaped in cross section, as in other enantiornithines [1]. The sternal half of the coracoid has a convex lateral margin and concave dorsal side (Fig 3), as in a number of other enantiornithines. The sternal width of the coracoid is approximately 45% of its length; this proportion is less than in *Eocathayornis walker* in which the sternal width is 50% the length of the bone. The scapular blade is largely straight, but it curves slightly along its distal fourth before tapering to a blunt end (Fig 3). The acromion process is well developed and costolaterally expanded; it forms a 150-degree angle with respect to the dorsal margin of the blade.

The cranial margin of the sternum is semicircular (Fig 3), resembling BMNHC-PH110 (holotype of “*Cathayornis chabuensis*”, regarded as a *nomen dubium* by Wang and Liu [10]) as well as the enantiornithines *Longipteryx chaoyangensis* [28], *Bohaiornis guoi* [29], and *Parabohaiornis martini* [30]. The morphology of this bone differs from the more acute cranial margin of *Cathayornis yandica*, *Houornis caudatus* and OFMB-3 (an indeterminate enantiornithine previously referred to as “*C*. *chabuensis*”; see [11]) (Fig 3). The overlap of the furcula obscures the craniocentral margin of the sternum, therefore the presence of a rostral spine cannot be determined (a condition present in *Eocathayornis walkeri* and *H*. *caudatus*; see [11]). Nonetheless, slab ‘b’ shows that the furcular hypocleidium fits into a deep trough developed on the cranioventral surface of the sternum, a condition considered an autapomorphy of *Junornis houi*.

The costal margin of the sternum, between the craniolateral corner and the base of the lateral trabecula, is generally straight (overlapped by sternal ribs on the right side of BMNHC-PH-919b). This condition resembles that of *Cathayornis yandica* and OFMB-3 (also that of enantiornithines such as *Bohaiornis guoi*, *Parabohaiornis martini*, and *Longipteryx chaoyangensis*), but contrasts with the varyingly concave margin of *Houornis caudatus*, *Eocathayornis walkeri*, and *Protopteryx fengningensis* (see [11]). BMNHC-PH-919a preserves a tiny process protruding from the craniolateral margin, at a point that is slightly more anterior than the base of the lateral trabecular (Fig 3). Similar small processes, although varying in morphology and placement, are known for some other enantiornithines (e.g., *C*. *yandica*, OFMB-3, *P*. *fengningensis*; see [11]).

The caudal margin of the sternum bears two pairs of trabeculae and a median xiphoid process (Fig 3), like in most enantiornithines [1]. The fan-shaped distal expansion of the lateral trabecula is slightly asymmetrical, projecting more medially than laterally. In this respect, *Junornis houi* appears more similar to *Eocathayornis walkeri*. Nonetheless, the shaft of the lateral trabecula is thicker in relation to that of the latter as well as with respect to *Cathayornis yandica* and *Houornis caudatus*. The intermediate trabeculae of BMNHC-PH-919 are fairly long (approximately half the length of the lateral trabeculae) and straight, a condition that contrasts with the short and medially curved morphology of these processes in *C*. *yandica* and *H*. *caudatus* (the relative length of the medial process stands out among other enantiornithines as well but is similar to the condition in OFMB-3). The xiphoid process is slender and reaches the same level as the lateral trabeculae; the lateral trabeculae extend further caudally than the xiphoid process in *H*. *caudatus* and *C*. *yandica*, but they are shorter than the latter process in OFMB-3.

#### Thoracic limb

**Fig 5:**
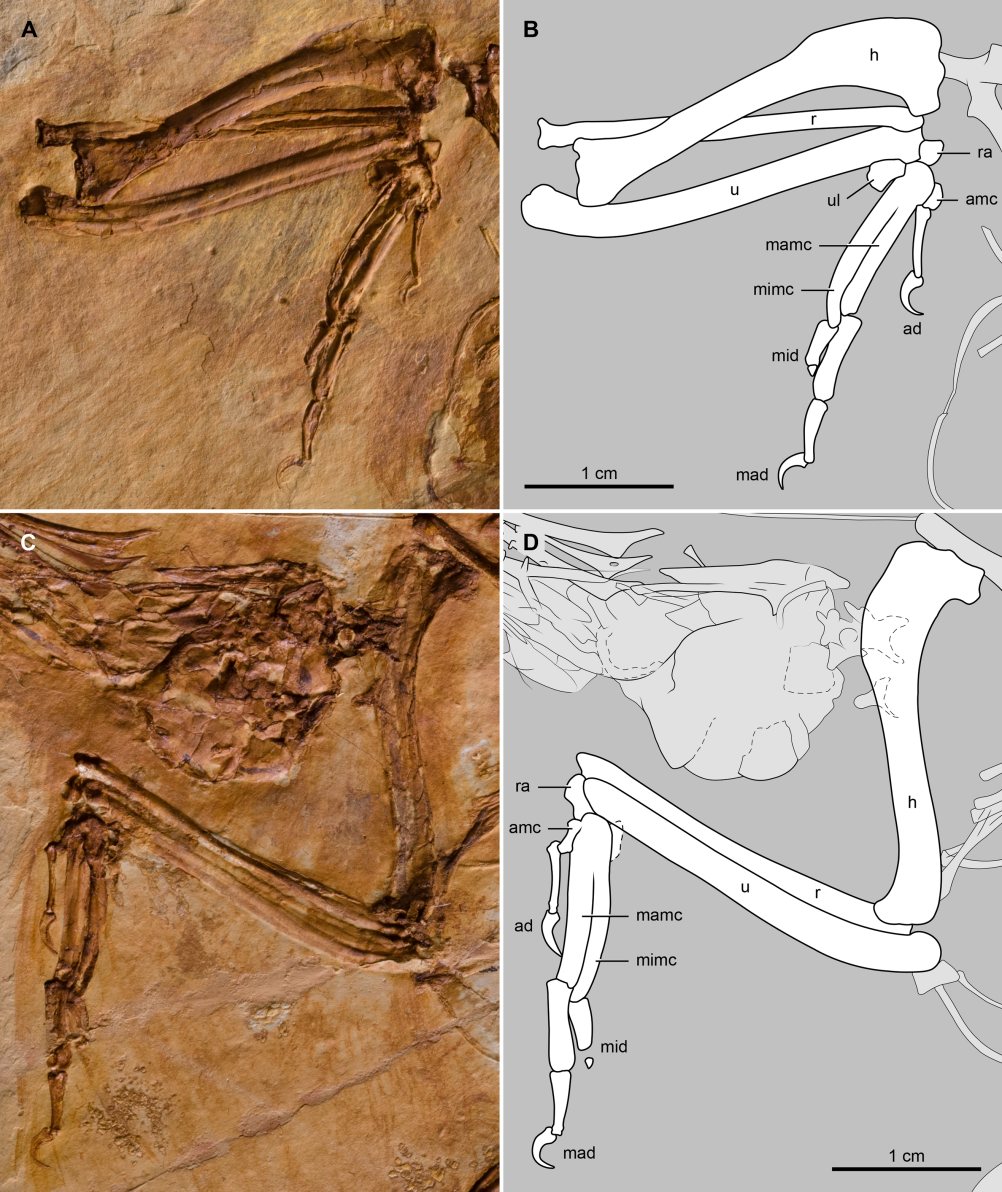
Photograph and interpretative drawing of the forelimb of *Junornis houi*. (A, B) BMNHC-PH919a and (C, D) BMNHC-PH919b. Abbreviations: ad, alular digit; amc, alular metacarpal; h, humerus; mad, major digit; mamc, major metacarpal; mid, minor digit; mimc, minor metacarpal; r, radius; ra, radiale; u, ulna; ul, ulnare.

The humerus is slightly shorter than the ulna (Table 1); its shaft is slightly sigmoidal. The contour of the proximal margin of the head resembles the ‘double hump’ morphology typical of enantiornithines [1] (Fig 5). The area of the bicipital crest is expanded, in agreement with *Cathayornis yandica* and *Eocathayornis walkeri*. The deltopectoral crest is narrower than the humeral shaft (Fig 5); it extends for approximately one-third the length of humerus as in *C*. *yandica* and many other Jehol enantiornithines [29]. At the distal end of the humerus, the flexor process is poorly developed, projecting slightly beyond the condyles. The ulna is slightly bowed along its proximal third but straight along the remaining portion of the bone (Fig 5). The radius is straight. The ratio of the ulnar/radial mid-shaft width is approximately 1.4; it is 1.6 in *Sinornis santensis*. As in *Houornis caudatus*, *C*. *yandica*, and *E*. *walkeri*, the ulnare is subrectangular (Fig 5), a morphology that differs from the more subtriangular contour (heart-shaped) of the ulnare in *S*. *santensis* [11]. The radiale is triangular and smaller than the ulnare, as in *E*. *walkeri*. The semilunate carpal appears fused or ankylosed with the major and minor metacarpals; the alular metacarpal seems fused with the major metacarpal as well. The short alular metacarpal has a rounded cranial margin (Fig 5A and 5B) as in many other enantiornithines (e.g., *Neuquenornis volans* [31]. The major metacarpal is straight and broader than the minor metacarpal. The intermetacarpal space between these bones is very narrow as in *C*. *yandica*, *H*. *caudatus*, and *E*. *walkeri*. The minor metacarpal is distally curved; it extends further distally than the major metacarpal, as in other enantiornithines [1] (Fig 5). The manual phalangeal formula is 2-3-2, as is common in most enantiornithines. The proximal phalanx of the alular digit is slender and slightly less than half the length of the major metacarpal. The ungual of this digit is relatively large, approximately half the length of the proximal phalanx (bony portion only). The proximal phalanx of the major digit is as wide as its metacarpal (Fig 5); it is the largest of all manual phalanges. The intermediate phalanx of this digit tapers slightly distally, as in *C*. *yandica* and *E*. *walkeri*; it is close to two-thirds the length of the proximal phalanx. The ungual of the major digit is slightly smaller than alular ungual. The proximal phalanx of the minor digit is slightly shorter than the intermediate phalanx of the major digit; distally, it articulates with a tiny second phalanx (Fig 5) that is rarely preserved in other enantiornithine fossils.

#### Pelvic girdle and limb

The preacetabular wing of the ilium, broader than its postacetabular counterpart, appears cranially rounded (Fig 4). The postacetabular wing is straight as in *C*. *yandica*; this condition contrasts that of *Houornis caudatus* and *Sinornis santensis* in which the postacetabular ilium develops a ventral curvature of varying degrees [11, 29, 32]. The pubic peduncle of the ilium is well developed, and directed caudoventrally, as in *Cathayornis yandica* and *H*. *caudatus* (it projects ventrally in *S*. *santensis*). The pubis is slender; the left and right elements define a broad (V-shaped) arc in ventrodorsal view (Fig 4). Considering the minimal distortion of the pubes and their natural articulation with other pelvic elements, the morphology of these bones indicates the presence of a broad pelvis (Fig 4). Distally, the pubes abut each other, as in all other enantiornithines [1]. The length of the ischium is more than half the length of the pubis. Proximally, the ischium exhibits a prominent dorsocaudal process as in many other enantiornithines [1] (Fig 4). The broad and subtriangular shape of this process, however, differs from the more slender morphology of the process in *S*. *santensis* [32] and some other enantiornithines.

**Fig 6:**
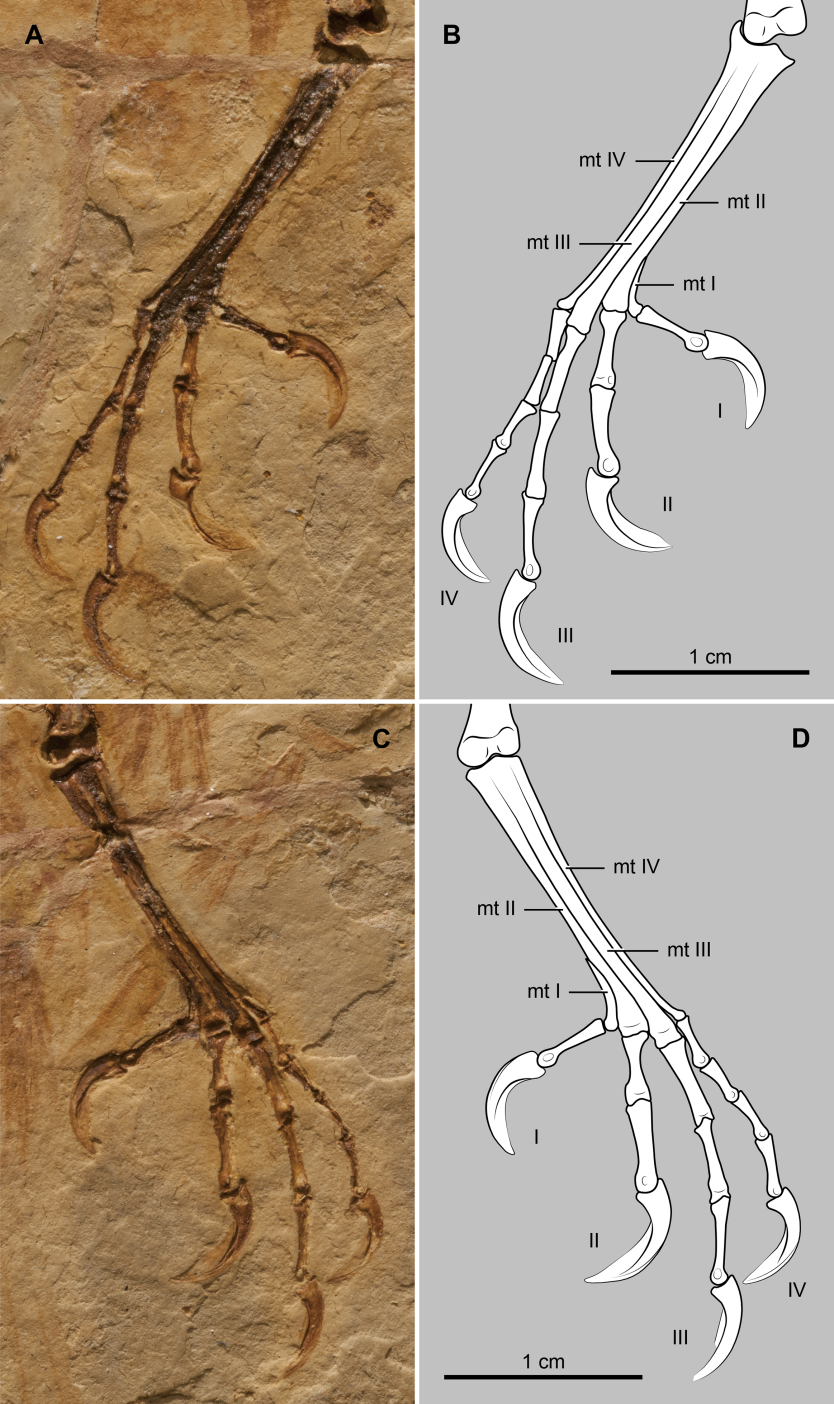
Photograph and interpretative drawing of the pes of *Junornis houi* (BMNHC-PH919a). Abbreviations: mt I-IV, metatarsal I-IV; I-IV, pedal digits I-IV.

The femur is gently bowed craniocaudally and approximately 83% the length of the tibiotarsus (Table 1). The proximal end of the tibia does not exhibit any development of a cnemial crest, a condition, typical of most enantiornithines [1]. Distally, it bears bulbous condyles of which the medial one is the largest. The slender fibula extends for about one-half of the tibia. Metatarsal III is the longest element of the foot; it is followed in length by metatarsals IV and II (Fig 6). Metatarsal IV is thinner than metatarsals II and III, as is common among enantiornithines [1]. Pedal digit III is the longest, but digit II is the stoutest (Fig 6). Digit I is opposable and about two-thirds the length of digit II, a pedal morphology that suggests perching capabilities. The bony claws of these digits are slightly curved and subequal in size.

#### Plumage

Contour, flight, and ornamental feathers are preserved as impressions (Fig 1). Non-pennaceous, contour feathers cover much of the skeleton except the wings and feet. Around the skull these feathers are slightly over 6 mm long. Longer feathers cover the pelvic region (8 mm average) and the neck (14 mm average). Contour feathers also cover the entire tibiotarsus forming ‘feather trousers’ common to many modern birds; their average length is about 11 mm.

While tightly packed, the preserved remiges define clear outlines of both wings, although the tip of the left wing appears to have been artificially added (Fig 1). Some of the longest primary feathers—measured from the distal portions of the major digit—are approximately 70 mm; the longest secondaries are about 71 mm long. Evidence of shafts and barbs are visible on some primaries and secondaries, although they cannot be sufficiently individualized as to permit analysis of barb-rachis attachment angles [33].

Two very long, tape-like ornamental rectrices project from the pygostyle in a V-shaped fashion (Fig 1); similar rachis-dominated feathers are known for a variety of enantiornithine birds including *Protopteryx fengningensis* [26], *Bohaiornis guoi* [29], *Dapingfangornis sentisorhinus* [34], and *Cratoavis cearensis* [35] among others [4–5]. The bases of these feathers, and the pygostyle, are surrounded by non-pennaceous contour feathers indicating the absence of pennaceous rectrices forming a caudal fan. The two ornamental feathers are about 200 mm long when measured from the tip of pygostyle. They have parallel margins and a faint median axis. Their tips are slightly expanded; the last 10% of the feathers show faint remnants of barbs, suggesting the development of vanes as the broad shaft would have tapered toward the feather’s tip.

All necessary permits were obtained for the described study, which complied with all relevant regulations. These were obtained from the Chinese government through the Beijing Museum of Natural History. All fossils and specimens collected by the Beijing Museum of Natural History, where the specimen is housed, conform to Chinese regulations.

## Discussion

BMNHC-PH 919 is considered to be an enantiornithine based on the presence of synapomorphies such as a ‘Y-’shaped furcula; a proximally forked pygostyle with ventrolateral processes; minor metacarpal projecting distally farther than major metacarpal; and a very slender metatarsal IV in comparison to metatarsals II and III [1]. The overall morphology and skeletal dimensions (Table 1) of BMNHC-PH 919 are similar to that of the holotypes of *Eocathayornis walkeri* [22], *Cathayornis yandica* [23], *Houornis caudatus* [24], and *Sinornis santensis* [32], “*Cathayornis chabuensis*” [9], and other small enantiornithines (e.g., OFMB-3). However, a combination of characters present in BMNHC-PH 919 indicates that this fossil belongs to a new taxon. This suite of morphological characters includes the presence of (1) a rounded craniolateral corner of the sternum (more angular or acute in *C*. *yandica*, *H*. *caudatus* and OFMB-3), (2) a distinct trough recessing the mediocranial region of the ventral surface of the sternum, (3) a triangular process developed in the base of the lateral trabecular of the sternum (absent in *H*. *caudatus* and *E*. *walkeri*), (4) a lateral trabecula that is significantly broader than those in *C*. *yandica*, *E*. *walkeri*, *H*. *caudatus*, and OFMB-3, (5) a deflected lateral trabecula (straight in *C*. *yandica* and *E*. *walkeri*), (6) a long intermediate trabecular that reaches close to the mid-shaft of the lateral trabecular (proportionally much shorter in *C*. *yandica*, *H*. *caudatus* and *E*. *walkeri*), (7) a xiphoid process level with the lateral trabecula (the trabecula extend further caudal in *H*. *cautus* and *C*. *yandica*), (8) strongly expanded costal process of the last penultimate synsacral vertebrae, which reach nearly 3 times the width of the same process of the last synsacral element, and (9) a very broad pelvis, based on the in situ placement of the proximal end of the pubes. Based on this unique combination of skeletal characters, we erect the new species, *Junornis houi*.

**Fig 7:**
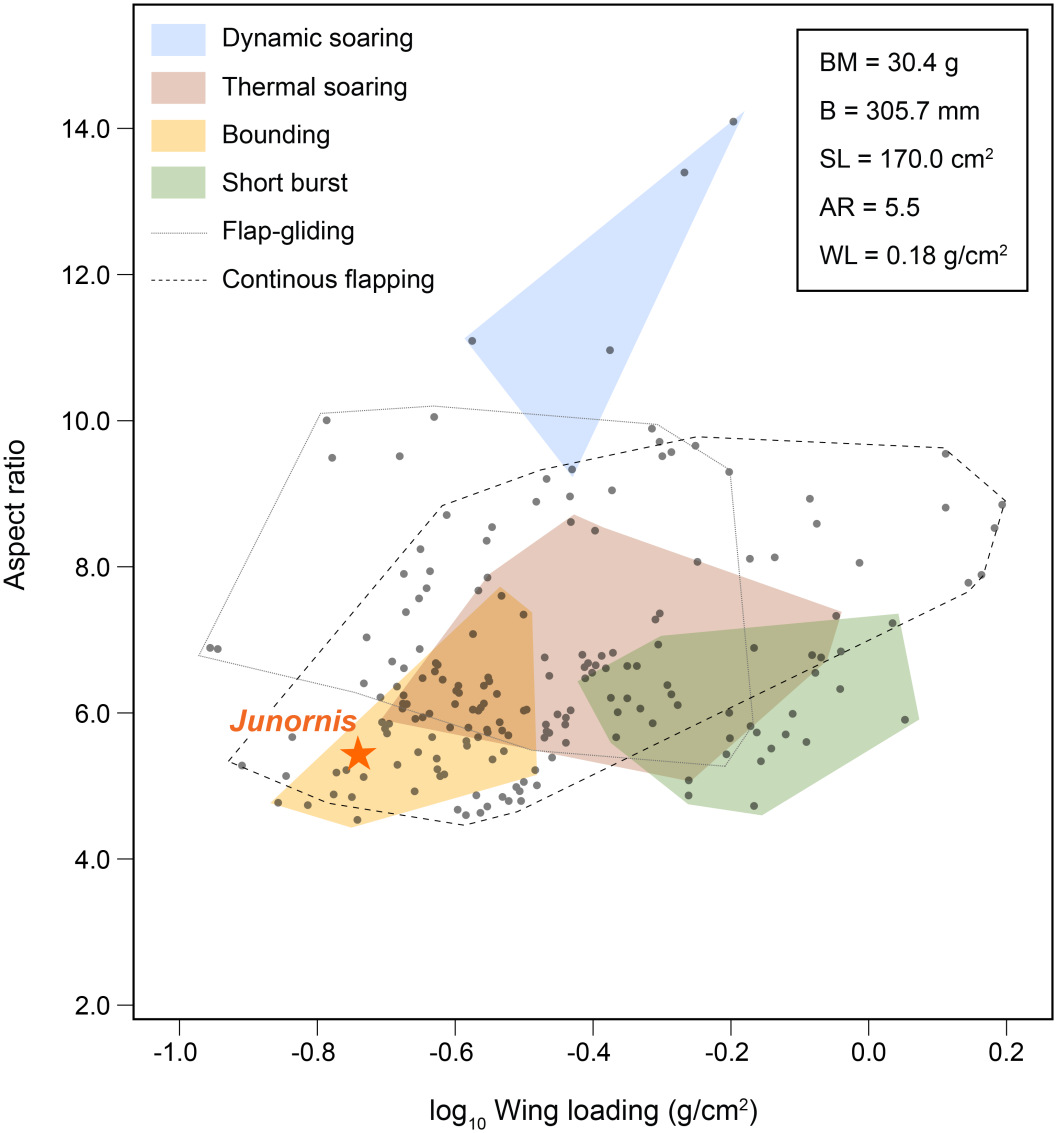
Morphospace of the flight modes of extant birds. Morphospace defined by the relationship between wing loading (*WL*) and aspect ratio (*AR*). The orange star points at the position of the holotype of *Junornis houi* (BMNHC-PH919). The values of the 188 modern specimens included in this analysis (gray points) come from Serrano et al. [13]. The inset lists the estimated values of body mass (*BM*), wingspan (*B*), lift surface (*SL*), aspect ratio (*AR*), and wing loading (*WL*) of BMNHC-PH919.

The well-preserved skeleton and exquisite plumage of BMNHC-PH 919 affords estimation of its flight capacity (see Methods). Using Serrano et al. [12, 13] quantitative models, we obtained values for the body mass (*BM* = 30.4 g), the wingspan (*B* = 305.7 mm), the lift surface (*SL* = 170.0 cm^2^), the aspect ratio (*AR* = 5.5), and the wing loading (*WL* = 0.18 g/cm2) of this fossil. These values indicate that the body and wings of this bird were similar, with the same low *AR* and *WL*, to those of modern passeriforms such as *Alauda arvensis* (Eurasian Skylark) and *Acrocephalus arundinaceus* (Great Reed Warbler) as well as other small-sized birds that fly using intermittent bounds (i.e., bounding) (Fig 7; see [13, 36–38]). As shown in Fig 7, the portion of the morphospace of *AR* and *WL* that is occupied by modern bounding fliers overlaps with the much broader portion of the morphospace occupied by continuous flappers (i.e., all birds that fly through intermittent bounds are also capable of continuous flapping) [13]. Like in modern bounding fliers, the low-*AR* wings of BMNHC-PH 919 could have generated large vortices at their tips that would have provided additional lift [39–43]. The low *AR* wings of BMNHC-PH 919 suggest that it may have been adapted to rapid take-offs, given that modern birds with proportionally short, broad wings (i.e., low AR) tend to maximize thrust during slow flight [44–45]. The low *WL* value of BMNHC-PH 919 indicates that this bird would have been able to generate a large magnitude of lift at low speeds because for a given speed and angle of attack, birds with greater wing area (and therefore lower *WL*) generate more lift than those with small wing areas [46]. Likewise, the low *WL* values of BMNHC-PH 919 suggest that this bird would have been highly maneuverable and able to perform tight turns (i.e., *WL* is proportional to turning radius) [47–49].

These inferred aerodynamic parameters indicate that BMNHC-PH 919 was an accomplished flier, a conclusion consistent with other lines of evidence [33, 50–51] indicating the same aerodynamic properties for small enantiornithines. However, the combination of small size, low *AR*, and low *WL* impose higher aerodynamic drags (i.e., induced and profile drag) [39–43, 52], and the values for induced drag and profile drag are known to increase with speed. Yet, when flying at higher speeds, *Junornis* could have reduced the cost of transport by switching from continuous flapping to an intermittent bounding flight. This strategy, used by most living passerines and woodpeckers, alternates continuous flapping with a ballistic phase in which the wings are folded against the body. Adopting a bounding flight strategy would have helped *Junornis* reduce the aerodynamic costs imposed by its size and wing shape, just as it does for its modern analogues [38, 43, 52–54]. While the mass-specific power available from flight muscles imposes an upper size limit to bounding flight [55], the estimated mass of *Junornis* (30.4 g) is significantly lower than the mass of the largest bird for which this flight mode has been described (i.e., Pileated Woodpecker at 260 g; [54]). Assuming a modern relation between body mass and flight muscles, the mass-specific power available from the flight muscles of BMNHC-PH 919 would have been well within the range where bounding flight is advantageous.

## Conclusions

The discovery of *Junornis houi*, the first published record of a Jehol enantiornithine from Inner Mongolia Autonomous Region, extends the geographic distribution of these early birds into the eastern portion of this region. The well-preserved wings and overall plumage of BMNHC-PH 919 adds significant information to the poor evidence of wing shape in Enantiornithes. Multiple regressions of skeletal elements and remiges allow estimation of key aerodynamic parameters (aspect ratio and wing loading) for this new enantiornithine. The small size, low aspect ratio, and low wing loading of BMNHC-PH 919 indicate that *Junornis houi* and other similar enantiornithines could have used bounding as their typical flight mode, especially at high speeds. The general morphology of BMNHC-PH 919 thus supports previous interpretations [13] indicating that most avian flight modes have very ancient origins; bounding flight might have evolved among enantiornithines more than 125 million years ago.
